# Agreement and Disparities between Women and Stop-Smoking Specialists about How to Promote Adherence to Nicotine Replacement Therapy in Pregnancy

**DOI:** 10.3390/ijerph18094673

**Published:** 2021-04-28

**Authors:** Lisa McDaid, Ross Thomson, Joanne Emery, Tim Coleman, Sue Cooper, Lucy Phillips, Felix Naughton

**Affiliations:** 1 Behavioral and Implementation Science Group, School of Health Sciences, University of East Anglia, Norwich NR4 7UL, UK; joanne.emery@uea.ac.uk (J.E.); f.naughton@uea.ac.uk (F.N.); 2 Division of Primary Care, University of Nottingham, Tower Building, University Park, Nottingham NG7 2RD, UK; ross.thomson1@nottingham.ac.uk (R.T.); tim.coleman@nottingham.ac.uk (T.C.); sue.cooper@nottingham.ac.uk (S.C.); lucy.phillips1@nottingham.ac.uk (L.P.)

**Keywords:** smoking cessation, pregnancy, nicotine replacement therapy (NRT), medication adherence

## Abstract

Evidence for the effectiveness of nicotine replacement therapy (NRT) for smoking-cessation in pregnancy is weak. This has been attributed to insufficient dosing and low adherence. This study investigated the acceptability of key messages and delivery modes for a behavioral intervention to increase NRT adherence in pregnancy. Semi-structured telephone interviews were carried out with pregnant or postpartum women aged ≥16 from across England, who had been offered NRT during pregnancy as part of a quit attempt and who struggled to quit (*n* = 10), and a focus group with stop-smoking specialists from across England (*n* = 6). The two data sources were coded separately using a thematic approach and then integrated to compare perspectives. Women and specialists agreed on message tone and delivery modes. However, views diverged on the most influential sources for certain messages and whether some information should be given proactively or reactively. There was also disagreement over which messages were novel and which were routinely delivered. This study demonstrates the value of capturing and integrating different perspectives and informational requirements when developing behavior-change interventions. The findings provide useful insights for designing a pregnancy-specific NRT adherence intervention that is acceptable to both those who will deliver and receive it.

## 1. Introduction

Smoking during pregnancy is the leading avoidable cause of poor birth outcomes, including risk of miscarriage, preterm birth, stillbirth, and low birthweight [1,2,3,4,5]. Smoking rates among pregnant women have been slowly declining in high-income countries but many women still find it difficult to stop; these women have an estimated prevalence of 8.1% in Europe and 5.9% in the Americas [6]. In England, the Department of Health and Social Care introduced a national ambition to reduce the rate of smoking in pregnancy from 10.7% in 2017 to no more than 6% by 2022 [7], but there has been very little change since then (9.9% in November 2020) [8]. The prevalence of smoking in pregnancy is significantly higher among disadvantaged groups [9,10,11]. These women are often dealing with multiple challenges and face barriers to accessing treatment services [12]. Reducing smoking in pregnancy is therefore a continuing priority and action is urgently needed to ensure that all pregnant women get the help they need to quit. 

National guidelines in the UK, Canada, Australia, and New Zealand recommend nicotine replacement therapy (NRT) for pregnant women who are unable to stop smoking with behavioral support alone [13,14,15,16]. There is no evidence to date that NRT causes harm during pregnancy but its effectiveness for smoking cessation in this population remains unclear: the 2020 Cochrane Review of nine placebo and non-placebo NRT trials found low-certainty evidence of a clinically significant improvement in smoking cessation rates. However, when only the six higher-quality placebo-controlled trials were included in the analysis, NRT was found to work no better than the placebo for improving smoking cessation in pregnancy [17]. There are two key factors that might explain this: (1) nicotine clearance appears faster in pregnancy, so a higher NRT dose is likely needed to alleviate withdrawal symptoms [18,19], (2) pregnant women can be reluctant to use NRT, and when they do, adherence is generally poor [17]. 

There are many different reasons pregnant women fail to adhere to NRT, some of which could be targeted by behavioral interventions. In line with the necessity–concerns framework [20], research has shown that women are worried about the safety of NRT in pregnancy. In particular, the concern is that it might increase their nicotine exposure compared to smoking, potentially causing more harm to the fetus [21]. Experiencing or anticipating negative side effects is also a major reason for NRT non-adherence and discontinuation [21,22]. Our own qualitative work has reinforced these findings and also revealed that some women believe NRT is unnecessary or ineffective, while others seek to test whether they still need NRT by stopping prematurely, which can leave them more vulnerable to relapse. Importantly, concerns about safety, nicotine intake, and nicotine dependency are often heightened in the context of combination NRT (using a patch plus a fast-acting NRT product) [23]. Healthcare professionals who deliver smoking-cessation advice can also be cautious about NRT use in pregnancy, especially when it comes to discussing topics such as safety [24,25] or use during a smoking lapse [26]. High-quality evidence on NRT use in pregnancy, and what works to help women use it properly, is therefore needed.

The N-READY (nicotine replacement effectiveness and delivery in pregnancy) program aims to develop and test a behavioral intervention, called “Baby, Me & NRT”, to improve adherence to NRT in pregnancy. This will enhance standard cessation advice in a stop-smoking service setting, by providing additional practitioner training and new information, educational materials, and tailored digital support for pregnant women. This qualitative study aimed to elicit women’s and stop-smoking specialists’ views on key intervention messages and how these might be delivered to assess and enhance acceptability. The findings will be used to specify the prototype NRT-adherence intervention prior to optimization and randomized controlled trial testing.

## 2. Materials and Methods

Semi-structured telephone interviews were conducted with women from across England who had been offered or accepted NRT as part of a quit attempt during pregnancy. We also ran a focus group with professionals who either directly supported pregnant women to stop smoking or were involved in the organization of stop-smoking support (referred to as ‘stop-smoking specialists’). Approval was granted by East Midlands—Nottingham 1 NHS Ethics Committee (reference: 12/EM/0388). We have adhered to the Consolidated Criteria for Reporting Qualitative Research (COREQ) checklist [27].

### 2.1. Public Involvement

A public involvement advisory panel (PIAP) made up of four women who have experience of smoking in pregnancy (*n* = 2) or an interest in the topic (*n* = 2) was established to offer insights into the N-READY program. Panel members were recruited through previous research with pregnant women, personal contacts, and word of mouth. For this sub-study, panel members were involved in the development of study and participant materials (e.g., topic guides for interviews/focus group, recruitment adverts, participant information sheets), the development of intervention messages, and reviewing the qualitative findings. 

### 2.2. Participants and Recruitment

#### 2.2.1. Interviews with Pregnant Women

We sought women who: were pregnant or had given birth in the last 6 months, were at least 16 years old, had tried to quit smoking while pregnant, and had been offered NRT to help them. Women were recruited via a paid Facebook advert campaign, which was run from our N-READY Facebook page. This allowed us to target women based on age and location. We set a maximum daily budget of £15 to manage the flow of women registering an interest in taking part. Those clicking on the advert were directed to an online registration form. To ensure that we captured the views of those who may find it hardest to quit, the advert was worded to purposively target women who had declined NRT, who had discontinued NRT prematurely or who had not cut out cigarettes completely whilst using NRT. As we aimed to provide NRT-related intervention messages to this specific population of women, which we have worked with before on NRT-related research [21,23], we did not anticipate requiring a large sample before saturation occurred.

#### 2.2.2. Focus Group with Stop-Smoking Specialists

Participants for the focus group were recruited via an email invitation sent to individuals involved in smoking cessation support for pregnant women in England, such as stop-smoking practitioners or service leads. The contact list was compiled from health professionals involved in a previous study who had agreed to take part in further research. We aimed to recruit between 6–8 participants, which is considered the optimal size to allow for rich discussion [28]. We anticipated fairly homogenous responses from our formative research with this participant group [29]. 

### 2.3. Procedure

As part of the intervention development process and informed by the theoretical domains framework (TDF) [30] and necessity–concerns framework [20], we developed a draft intervention structure with a set of NRT-related key messages, which might be used to address pregnant women’s behavior. These were either novel or already part of the English National Centre for Smoking Cessation and Training’s (NCSCT’s) standard treatment program for pregnant women [31] but considered essential for NRT adherence. The messages were structured around seven themes: (1) NRT can help you quit, (2) NRT is safe to use in pregnancy, (3) the importance of taking NRT as instructed, (4) tips for taking NRT, (5) identifying and managing withdrawal symptoms and side effects, (6) NRT use in a lapse, and (7) getting others on board with your quit attempt. The qualitative data collection with women and specialists sought feedback on a selection of these key intervention messages. The example messages presented to each participant group were not identical, but there were eight similar issues associated with NRT use in pregnancy and adherence to NRT discussed with both (see Table 1). 

For the interviews with women, messages were emailed to participants beforehand. A semi-structured topic guide was then used to identify what, if anything, would make them more effective and improve acceptability. We also asked what participants considered to be the best ways for these messages to be delivered. Verbal consent was obtained at the beginning of the interview and a hard copy was sent to the women afterwards, along with a £20 shopping voucher as a thank-you for taking part. The interviews took place between January 2019 and March 2019, lasted an average of 67 min, and were conducted by LM (PhD). 

Focus group participants were shown PowerPoint slides that described the NRT adherence-related issue to be addressed, example messages, and prompts to aid discussion. The focus group took place at the University of Nottingham in February 2019, lasted three hours, and was conducted by RT (PhD). Written consent was obtained on the day. Both LM and RT are trained in and have considerable experience in conducting and analyzing qualitative research.

### 2.4. Analysis

As the interviews and focus groups involved different topic guides, and the way the intervention messages were presented to participants differed, these data were initially coded separately. Feedback included whether message responses were positive or negative, the perceived novelty of the messages, and thoughts on by whom, how, and when the messages might be delivered. We also sought to identify any new emergent themes from the data that might help with refining the adherence intervention as a whole. 

To begin, audio recordings were transcribed verbatim and a process of familiarization was undertaken by reading each transcript and making notes. The intervention messages in each topic guide informed the initial thematic frameworks. Nvivo 11 software was used to facilitate data management, with coding undertaken independently by two researchers. A summary of the key findings was then produced for both the interviews and the focus group with suggestions for refinements to the protype intervention. 

For a robust comparison of findings from the women’s interviews and the specialists’ focus group, we identified the issues and associated intervention messages that were discussed across both. LM and RT then reviewed the summarized data relating to each of these, along with views on delivery, to look for similarities and differences in perspectives between the pregnant women and the specialists. Subsequent discussions identified three themes that best explained the data (see Supplementary Figure S1). These were then used as the framework for producing a comparison table. The findings from the comparison exercise were discussed with the entire research team and our public involvement advisory panel, to provide complementary perspectives.

## 3. Results

Thirty women registered an interest in the study, of which 10 were contactable and consented to take part. Two women were using NRT and smoking, five had used NRT but relapsed to smoking, one had used NRT but stopped and quit smoking unassisted, and two had turned down the offer of NRT. Participants were aged between 16–39, the majority were white British, and two thirds (*n* = 7) were living with a partner. Two were in the first trimester (≤12 weeks gestation), four were in the second trimester (13–26 weeks), two were in the third trimester (27–40 weeks), and two were in the postpartum period (0–6 months) (see Table 2). The cost for recruitment was £11.96 per participant. 

The focus group consisted of six participants who were either stop-smoking practitioners (*n* = 3) or worked in the organization of stop-smoking-in-pregnancy services (*n* = 3) (see Table 3). All came from different services from across England. Not all invited specialists were able to participate due to their workload. The findings were organized around three themes: (1) views on the novelty, value, and delivery timing of key intervention messages, (2) message source and communication style, and (3) preferred modes of delivery. Brief summaries and representative quotes for each theme are used for reporting purposes. Quotes from participants are identified in the following manner: participant 1 from interviews (P1 Int), participant 1 from focus group (P1 FG).

### 3.1. Views on the Novelty, Value, and Delivery Timing of Key Intervention Messages

Within this theme, we look at each of the eight NRT adherence-related issues and examine women’s and specialists’ views on the example messages designed to address them in terms of novelty, informational preferences, and ideal message timing.

#### 3.1.1. Addressing Concerns about NRT Safety (Issue 1)

Both women and specialists felt that emphasizing the safety of NRT was very important. However, specialists were unsure about saying NRT was ‘safe’ in pregnancy because in their view it was not completely risk-free. On the other hand, pregnant women thought it was most important to focus on communicating the health benefits of using NRT rather than talking about the risks, particularly for the baby, but they also wanted to know how much safer NRT was when compared to smoking. It was suggested that this message would be more persuasive and memorable if it stated an estimate of risk in terms of how much safer NRT was: “*More facts. State, like, how is it better? I mean, what percentage?*” (P9 Int).

#### 3.1.2. Awareness That Nicotine Is Metabolized Faster in Pregnancy (Issue 2)

Interviewed women thought that the message they were presented with about increased nicotine metabolism in pregnancy was very useful and should be given early on, as it helped to explain why pregnant women might experience stronger nicotine cravings and contextualized the need for higher NRT doses. However, very few women who received NRT advice recalled being told this:

*I didn’t know that. I’m a bit shocked by that, to be fair, because obviously I’ve been to see somebody about stopping smoking and I didn’t get told that.*

(P2 Int)

In contrast, specialists perceived that this type of message would already be routinely given during the first consultation but emphasized the need to deliver it in an understandable way to have the intended impact: “*If you explain it right, it lands quite well*” (P1 FG).

#### 3.1.3. Addressing Concerns about Nicotine Exposure and NRT Dependency (Issues 3 and 4)

Women had concerns about nicotine and getting too much of it, even after receiving the message that this was not the harmful substance in cigarettes. The message about getting no more nicotine from combination NRT than from smoking tobacco was said to be persuasive in terms of encouraging the use of two NRT products together and easing concerns about nicotine exposure from NRT. However, it was not something any of the women said they had heard before:

*No, I just assume using two products would be double the amount of nicotine, so more than what I’d be using in the first place. But to know that wouldn’t be the case [would be good], I bet quite a few people think the same thing.*

(P8 Int)

Specialists reported liking the message; however, they indicated that it was not information women needed to be told at the outset but could be discussed if and when it came up in follow-up consultations. The stop-smoking practitioners in the focus group said that they would typically talk about getting clean nicotine from NRT rather than comparing different levels of nicotine intake between NRT and smoking. If fact, they felt that women were probably more worried about not getting enough nicotine:

*I think again you’re down to having that conversation when that lady asks you, or if that lady’s worried about having too much nicotine, then you can go into that conversation. It’s not a sort of thing I’d be telling them right at the beginning.*

(P4 FG)

*If a woman had voiced a concern, then I would deliver this message but in my experience the concern is more about it not being enough.*

(P2 FG)

On the theme of nicotine, pregnant women also found the message that NRT is less addictive than smoking reassuring, as for many, this thought had crossed their minds. It was considered important to explain why this was the case. One woman suggested that using NRT could be described as a nicotine “*reduction program*” (P6 Int). Again, specialists only thought this message should be discussed if it was raised by a client:

*I think that would come later on, you know, if they are becoming dependent on something. I don’t think it’s something that I’d talk about straight away.*

(P3 FG)

#### 3.1.4. NRT Can Be Used throughout Pregnancy (Issue 5)

Most women thought that knowing NRT can be used throughout pregnancy and beyond was useful, but this was not something they reported having heard before. Some of those women who had received NRT advice described not being told how long they should use NRT for at all: “*Advisor did not indicate how long I would need to use NRT for.*” (P2 Int).

Specialists liked this message too, and some said that it was already being delivered. However, the main concern with this message was whether commissioning would restrict services from offering longer courses of NRT:

*It depends on how you’re wording that message because it is OK for them to stay on NRT throughout their pregnancy, but we don’t want to give them the assumption that we’re going to give it to them, because we can’t. And different areas have different budget constraints, so it might be worth having that caveat…*

(P2 FG)

Specialists thought that explaining NRT can be used throughout pregnancy may not be discussed until a follow-up appointment, and as part of this conversation, it would be important to discuss how doses of NRT will be gradually reduced over time. 

#### 3.1.5. Continuing to Use NRT in a Lapse (Issue 6)

The majority of pregnant women were very receptive to the message they received on continuing NRT use during a lapse, as they would count a lapse (smoking one or more cigarettes, but not totally relapsing to smoking) as failing and, so, some foresaw themselves stopping NRT altogether in this situation. They believed that if women were given this information from the outset, this would help them feel less like a failure and encourage them to continue with their quit attempt:

*Well, I think that would be good because without being told that, I’d think, “Oh well, now I’ve had a cigarette I can’t have those patches and then I’ve failed so I need to stop with the patches,” or something.*

(P5 Int)

*I would think that—I think that’s good and is encouraging in a way that it means “OK, I mucked up, but don’t lose the hope.”*

(P8 Int)

A few, however, were concerned about continuing to use NRT during a brief lapse; they thought that they might take in too much nicotine, which could harm the baby and, so, wanted more information about nicotine intake. Indeed, this concern had led some of the women interviewed to alternate between NRT use and smoking:

P: *I think more information on how much nicotine you’re taking […] So, I had the patches and then I’d take off the patch, have a fag, and then I tried to go back to it but then that weren’t helpful either really, to be honest.*

I: *So, you’d take off the patch when you had the cigarette?*

P: *I think because I thought I’d be taking in too much nicotine.*

(P7 Int)

However, interestingly, when specialists were shown a message which might address these concerns, which explained that if pregnant women use NRT and smoke they will probably be exposed to no more nicotine than smoking alone, most felt “uncomfortable” or “didn’t agree” with the message:

*I feel quite uncomfortable with it. I’d probably need to see more in the way of evidence because it seems to go against everything that we’ve done previously.*

(P2 FG)

*I think this research is good for us to know but we really don’t need to be telling anybody else about it.*

(P4 FG)

Some of the women interviewed thought that being told to continue NRT in a short smoking-lapse period might encourage pregnant women to think it is acceptable to smoke and use NRT generally. Likewise, most specialists thought that this message might give women permission to fail, so did not think women should be given this information upfront:

*I think this would be quite a difficult message to give. It’s one of those where you don’t kind of want to give it because it’s almost like saying “Oh, you might smoke a bit”. It’s almost pre-empting that they are going to slip up.*

(P5 FG)

*…[it might give them] that confidence to think “Well, I can just have a cigarette whenever I want one” and that’s when you start sliding down the slippery slope again.*

(P3 FG)

Although they did appreciate that there may be instances where this information could be useful:

*I think it’s a message for us to know and for us to use if needs be.*

(P4 FG)

*… It’s more reactive as opposed to proactive.*

(P1 FG)

#### 3.1.6. Don’t Stop NRT Too Early (Issue 7)

Both women and specialists thought the messages relating to prematurely stopping NRT were positive and agreed that women should be given this information in the first consultation. A number of the women drew parallels with antibiotics, where the advice is to take the full prescribed course of treatment. Similarly, one specialist likened NRT to using painkillers: “*You don’t know that that pain’s there until you stop taking them, so you’re sort of fooled into thinking that you’re OK*” (P2 FG). While another said that this message tied in well with explaining the duration of treatment.

#### 3.1.7. Addressing Concerns about Side Effects (Issue 8)

Both women and specialists thought that it was a good idea to refer to NRT as a “medicine”. Some specialists were concerned about suggesting side effects were likely to be temporary and “usually disappear” as the term “usually” could be off-putting: *“… they usually disappear? I suppose if I read that I’d be picking up on “usually”.*” (P2 FG). However, women found this information reassuring. 

Specialists were also uncertain about discussing side effects at the first consultation because this may give women an excuse as to why they do not want to continue with NRT:

*Sometimes these are reasons why women then decide not to carry on. I really think it’s about boosting their ability to quit and if you tell them that they might get all these things, “I’m not going to bother, then”.*

(P3 FG)

However, side effects might be discussed upfront if it came up during a conversation about previous NRT use: “*Then quite often it’s, “Oh, I couldn’t use them because my arm comes up”, or, “I’ve tried this”.*” (P1 FG). On the other hand, women felt it was important to establish expectations at the outset of treatment and to know that there are ways to minimize side effects. For some, this might have made them persevere with taking NRT for longer:

*I think so, because, obviously, where I was feeling sick, I think if I’d have heard that or been told that then I may have stuck it out a bit longer.*

(P4 Int)

### 3.2. Message Source and Communication Style

When asked about the most influential sources for different messages, women indicated that they would like real-life stories when receiving information about the harms of smoking in pregnancy or insights about using NRT. For example, one participant said “…*I mean, they’ve gone through the same thing—the irritation [with a patch]—so if there’s a way they can help someone else stop that, it would encourage them to carry on*” (P9 Int). Women also wanted to hear “realistic” accounts from former pregnant smokers who had quit with NRT:

*Because you find that a lot with the professionals and things—yes, obviously, they know more about it but they read from a textbook, you know, and to have somebody that’s in your situation or been in your situation and knows what you’re going through and seeing they’ve come out the other side would be very helpful.*

(P2 Int)

*You want to hear of people who have started and probably struggled. It would probably help more if they’ve struggled, gone on their own and then gone back to it and actually finished the course [of NRT], proved themselves wrong, but proved that they’ve got to finish the course for it to work properly.*

(P9 Int)

Specialists recognized that the experiences of others, such as friends and family, could be influential, but also cautioned that this was often a source of misinformation.

*A lot of the mums we see, you know, whatever their friends say or their mum’s said, you know, if they’ve heard that first then, like, it is hard to overcome that, isn’t it? It’s doable but you do need to get that rapport with somebody.*

(P5 FG)

Women expressed an interest in hearing “facts” and “research” for more medical-related issues, such as NRT safety in pregnancy, the likelihood of long-term addiction to NRT or continuing to use NRT in a brief lapse. This information was considered best delivered by health professionals or other leading health experts:

*Dealing with these concerns that people might have, to have some sort of expert, like authority on nicotine replacement therapy in pregnancy to, sort of, be saying, ‘This is, sort of, the most up to date, what we know’.*

(P4 Int)

*Cite research—that’s what you need, straight to the point, you know, that’s what you need to be able to just know that and then that can put people’s minds at ease.*

(P2 Int)

However, specialists were of the opinion that pregnant women would not be particularly interested in listening to academics or talk about “scientific” information:

*I think it should be a mix of midwives and Stop-Smoking Advisors rather than Professor X […] and speaking about scientific findings, I’m not sure that the women I work with would be particularly [persuaded].*

(P2 FG)

Both specialists and women agreed on the importance of consistency and keeping messages simple: “*…you just want a load of clear, key points and you know what you’re doing*” (P9 Int). In terms of message tone, pregnant women indicated that messages should be supportive and encouraging:

*I just think support and encouragement when you’re trying to do something like that is what you need.*

(P2 Int)

Which was echoed by specialists:

*And you want to give them as much positivity and encouragement and confidence that they’re going to be fine, you know.*

(P6 FG)

### 3.3. Preferred Message Delivery Modes

Interviewed women and stop-smoking specialists both said that it might be advantageous to use different delivery modes to ensure that pregnant women receive messages in an appropriate manner. This could include a combination of practitioner support, along with digital solutions such as a website, text messages, videos, and/or an app. There was recognition that people have different learning styles and that repetition across different media might be advantageous:

*It wouldn’t hurt for it to be heard more than once, definitely, like anything. And also, you know, to hear it perhaps in a different form rather than just verbal because some people don’t hear you talking, they might need to see it written down or something.*

(P4 FG)

*…some videos and stuff like that. Because videos always make—well, I prefer to watch videos than actually read. And it would just be good to get some of the information across. Especially people’s personal views.*

(P4 Int)

Specialists thought that a website would be a good place to emphasize key content and include information that they might omit from the first consultation:

*I think where it would have a place is if you had something like a website with myths and things like that. Myth—you will become addicted to NRT—false, blah, blah, blah. So that’s where it would have a place so it can be addressed as a sort of myth-buster.*

(P1 FG)

*If they were experiencing something [side effect] that they could then look up because that’s what they do, they phone instead of looking it up first.*

(P3)

Specialists reported a lack of trusted resources that were relevant to using NRT in pregnancy but, although a dedicated website seemed like a good idea, they cautioned that an app might be more suited to the target population:

*I think an app would also be more accessible to more women than a website specifically because you’ve got to think about the age group of people getting pregnant and particularly the younger generation… I know you can get websites on your phone but it’s more user-friendly in an app form.*

(P1 FG)

A number of pregnant women also suggested that “*online forum where people can all talk together*” (P9 Int) might be useful, but this was not raised in the specialist focus group. 

## 4. Discussion

This qualitative study identified similarities and differences in the views of women and specialists on key messages for an intervention to improve adherence to NRT in pregnancy. There was general agreement in terms of message tone and the best methods for delivery. However, women and specialists had differing views on whether or not messages were novel, the most influential sources for certain messages, how best to communicate research evidence, and the timing of message delivery. These findings demonstrate the value of capturing and integrating different perspectives and informational requirements when developing behavior-change interventions. 

In this study, specialists were sometimes resistant to proactively discussing women’s concerns about NRT or to provide them with information to cope with an issue before it happened. This particularly related to potential side effects, nicotine intake, and continuing NRT use in a smoking lapse. Evidence has shown that health professionals might contribute to poor adherence by withholding information because they think it will deter patients from taking medication [32], and that positively framed risk information might help improve use [33,34]. Meanwhile, on the other hand, patients often voice frustration about not receiving as much information about medication side-effects or risks as they would have liked, believing that full disclosure would help them make more informed treatment decisions [35]. A recent qualitative study that focused on how women make decisions about medication in pregnancy found that many of the women wanted to move beyond the basics and know about the risks and benefits of medication use upfront. Not only can this support informed choice, but having realistic perceptions might help to reduce anxieties [36]. The women in our study seemed particularly interested in knowing what to do in a smoking lapse, but specialists had strong concerns about recommending continued NRT use in this situation. Identifying what constitutes a lapse, as opposed to a full relapse, and the appropriate management with NRT in this population, are matters that warrant further research [26].

We also identified specialist uncertainty about what could be said in relation to NRT in pregnancy. Yet most women felt they needed clear and unequivocal information to help them make decisions about NRT use. For example, to offer an estimate of how much safer NRT is than smoking might be particularly memorable and persuasive. Practitioner uncertainty can often arise from a lack of knowledge or lack of evidence [37] and can result in inconsistent information provision. Specialist training for practitioners could give them the knowledge and confidence to deliver enhanced NRT support in pregnancy and a stronger evidence base regarding exposure to nicotine from smoking and NRT could help inform such training.

In terms of information sources, women thought that messages on the consequences of smoking in pregnancy and using NRT were considered more persuasive if they came from another’s personal experience. Hearing stories from women who have successfully quit smoking, and the opportunity to communicate with those also trying to quit, has previously been identified by pregnant women as a useful feature in smoking cessation interventions [38,39]. Indeed, Coley et al. [40] argued that smoker-informed content is more realistic to experience and therefore it may help to maintain engagement with interventions. However, the effect of peer interventions on smoking cessation outcomes in pregnancy is unclear [41,42] and they can be difficult to implement. This may be an opportunity for practitioner storytelling, using anonymous anecdotes or sharing videos or case studies of other pregnant women’s quit attempts with NRT; however, there is limited literature looking at the effects of this on smoking cessation. Online social networks have grown in popularity, and there is some emerging evidence that these have a positive effect on health-behavior change in the general population [43,44] with studies focusing on smoking cessation showing a moderately beneficial effect [45,46]. Further research is needed that specifically focuses on pregnant women, and to establish what role this type of support might play as part of a broader behavioral intervention. As noted by specialists in this study, the potential risks that arise from peer-to-peer communication also need to be carefully considered, as careful training and/or moderation is required, where appropriate, to minimize the spread of misinformation or judgement [47].

Using ‘credible experts’ to deliver support for smoking cessation in pregnancy has been identified as a common behavior-change technique in effective interventions [48]. Our findings also emphasized the need for evidence-based information from experts, which for women included health professionals or individuals with authoritative knowledge. Indeed, the feedback from women suggested that talk about ‘evidence’ and ‘research findings’ was more familiar and compelling than the specialists thought it would be. While some argue that there is not a one-size-fits-all approach to effectively discussing relevant information about the risks and benefits of a medical treatment [49], creating simple standardized text in collaboration with pregnant women might be a helpful way for stop-smoking practitioners to improve communication of the latest evidence-based information. Using the ‘teach-back’ method, whereby health professionals check they have explained information in a way that has been understood, might also be a helpful approach [50].

Some messages came as a surprise to pregnant women, such as increased nicotine metabolism during pregnancy, even though specialists perceived that these were routinely delivered. This could highlight women’s poor recall of particular information, with the effects of stress and anxiety thought to have a detrimental effect on memory performance in the clinical setting [51]. However, another important possibility regarding the perceived novelty of certain intervention messages might be differences in provider time and competence. Studies exploring the fidelity of NHS behavioral support for smoking cessation have shown inconsistency between services, with some studies showing that around one third of recommended content is typically not delivered [52,53]. This essential content may therefore need reinforcing via further in-services training or continuing professional education with practitioners to help maintain essential competencies and improve NRT adherence. A consultation checklist might also ensure that key messages to promote NRT adherence are not forgotten. 

When designing and developing behavior-change interventions, it is not only important to understand the techniques and mechanisms that lead to change but also the factors which might influence implementation in real-world settings [54]. The involvement of health professionals and service users in that process is both important and challenging when developing complex interventions [55], and taking into account the views of both of these stakeholders identified areas of consideration that may not have been discovered by consulting with either group exclusively. Consensus between the groups allowed us to identify areas of content tone and delivery that could be strengthened in any new intervention. However, divergent views between the groups were arguably more informative as they identified areas where there may be a need for increased focus (e.g., around peripheral treatment advice) or the open discussion around potentially sensitive subjects (e.g., treatment side effects). 

## 5. Strengths and Limitations 

Strengths of the work include the integration of women’s and specialists’ perspectives and the use of intervention messages informed by existing evidence and theory to stimulate feedback and discussion. However, this study has a number of limitations. The pregnant women interviewed did not attend the same services that the specialists represented. Given the variety of stop-smoking service models and contexts of delivery, this may explain why there were differences between the information women said they wanted and was new to them, versus what specialists believed was already delivered. Nevertheless, by recruiting a diverse sample we were able to highlight potential delivery gaps. While the focus group had sufficient participants to facilitate a blend of perspectives and encourage candid responses, specialists were only able to talk about their own practice and service. Caution, therefore, must be taken when interpreting the findings as they only represent the views of a specific group. Moreover, we recognize that due to changes in commissioning [56] and budgetary constraints, there are different models for delivering stop-smoking support in pregnancy. Therefore, the findings may not be representative of England as a whole. This also applies to the interviews with women, as the sample was predominantly composed of white British women. However, the online recruitment method facilitated engagement with women from disadvantaged backgrounds, who often disengage with stop-smoking services and may be missed using service-based recruitment. A drawback to using telephone interviews is that visual aids cannot be used to prompt discussion. Even though women were sent example messages in advance, we cannot be certain that they had read them. When considering different intervention messages and delivery modes it may be useful to show examples during the interview and use a ‘think aloud’ approach [57]. There was insufficient time to undertake any repeat interviews or participant checking, due to constraints in the timetable of the overall study. However, findings from the comparison exercise were discussed with our PIAP, which helped to provide another perspective on the analysis and shape the conclusions.

## 6. Conclusions

The findings highlight how women and stop-smoking specialists can have differing views on the acceptability, delivery, and content of key intervention messages to promote adherence to NRT in pregnancy. Views differed on how best to communicate key messages, with specialists particularly cautious about imparting some information upfront, such as about side effects or handling a lapse contrary to the women’s views. However, a number of novel or not yet routinely delivered messages about NRT use in pregnancy were liked by both groups of participants. The present findings highlight the importance of taking into account the perspectives of both those delivering and receiving a behavioral intervention at the development stage, in order to improve acceptability and feasibility.

## Figures and Tables

**Table 1:** Issues to address and associated intervention messages discussed in the interviews with women and the focus group with stop-smoking specialists.

	Issue to Address	Interviews with Pregnant and Postpartum Women Example Messages	Focus Group with Stop-Smoking Specialists Example Messages
1	Concerns about NRT safety	NRT is much safer than smoking because it doesn’t contain all the harmful chemicals you get from smoke, and these are the things that cause the harm.	NRT has never been shown to cause harm to babies.
2	Awareness nicotine is metabolized faster in pregnancy	Nicotine is processed more quickly by the body during pregnancy and therefore higher doses of NRT may be needed to manage withdrawal symptoms.	Nicotine is removed from your body a lot more quickly when you are pregnant; this means you need higher doses of nicotine to prevent cravings.
3	Address concerns about nicotine 1 (too much nicotine)	With combination NRT you are unlikely to receive doses of nicotine that are higher than you would receive from tobacco use.	NRT gives you a lot less nicotine than you would have received by smoking.
4	Addressing concerns about nicotine 2 (NRT dependency)	Using NRT is not trading one nicotine addiction for another. The way NRT is delivered makes it much less addictive than smoking and long-term dependence on NRT is highly unlikely.	Using NRT is not trading one nicotine addiction for another. The way NRT is delivered makes it much less addictive than smoking and long-term dependence on NRT, even in high doses, is highly unlikely.
5	NRT can be used throughout pregnancy	It’s OK to use NRT throughout pregnancy if instructed, as this will be safer than going back to smoking.	It is OK to use NRT throughout your pregnancy.
6	Continuing use of NRT during a lapse	If you were told to keep on using NRT even if you were smoking a little (as a means to helping you return to not smoking at all), what would you think?	If you do start smoking for a short time do not stop using the NRT (even if you continue to smoke a little). Re-commit to stopping and you can get back on track and stop smoking. Or: Research shows that if you try stopping smoking with NRT but lapse and smoke a little, the baby will be exposed to less tobacco smoke and no more nicotine than just smoking.
7	Don’t stop NRT too early	Don’t decide to take NRT depending on how you’re feeling—it is important to take the whole course for as long and as regularly as instructed, regardless of withdrawal symptoms or how confident about quitting you are feeling.	You can’t easily tell when NRT is working. If NRT is doing its job then you probably won’t notice it but don’t let this trick you into thinking you’ve quit already and you don’t need to keep taking it. It’s important to take the NRT for as long as recommended and don’t stop your NRT until you have spoken to your stop-smoking practitioner.
8	Addressing concerns about side effects	Side effects are typically mild, don’t get worse, and usually disappear—not a sign of anything bad to come. Or: Side effects can usually be managed using tips and tricks.	Like medicines, NRT products can have side-effects but they are typically mild, don’t get worse, and usually disappear. They are not a sign of anything bad. Or: The side effects of NRT are dizziness, headache, excessive sweating, nausea, palpitations, skin reactions, vomiting. You may experience these if you use too much NRT but this is very unlikely.

**Table 2:** Characteristics of pregnant and postpartum (PP) women.

ID	Age	Weeks Pregnant	Ethnicity	NRT-Related Experience
P1 Int	16	11 weeks	White British	Offered NRT but did not follow up. Having the occasional puff on a cigarette.
P2 Int	29	9 weeks	White British	Using nicotine patch. Smoking up to 5 cigarettes per day.
P3 Int	29	13 weeks	White British	Using nicotine gum intermittently/smoking 4–5 cigarettes per day. Occasionally using a vape.
P4 Int	17	38 weeks	White British	Used nicotine patch and inhalator for a few weeks but now relapsed. Smoking 15 cigarettes per day.
P5 Int	18	20 weeks	White British	Used nicotine patch <1 week (purchased herself). Smoking 2–3 cigarettes per day.
P6 Int	34	15 weeks	White British	Used inhalator and nicotine gum for about 6 weeks but stopped due to side effects. Continued quit attempt alone and now nicotine free.
P7 Int	24	27 weeks	White British	Used nicotine patch and mints briefly but stopped. Smoking 10 cigarettes per day.
P8 Int	39	21 weeks	Mixed White/Black Caribbean	Used nicotine patch and mouth spray but stopped due to side effects. Still smoking occasionally.
P9 Int	29	26 weeks PP	White British	Used nicotine patch and mints, then tried the mouth spray. Stopped NRT as did not feel it was working. Now smoking 10 cigarettes per day but was 2 per day at the end of pregnancy.
P10 Int	2 6	17 weeks PP	White British	Offered NRT but did not accept. Concerns about using in pregnancy. Has continued to smoke around 10 cigarettes per day.

**Table 3:** Characteristics of stop-smoking specialists.

ID	Age	Gender	Job Title	Experience
P1 FG	61	F	Smoking cessation midwifery lead	>10 years
P2 FG	45	F	Stop-smoking-in-pregnancy specialist trainer	>10 years
P3 FG	40	F	Stop-smoking practitioner	2–5 years
P4 FG	48	F	Stop-smoking practitioner	2–5 years
P5 FG	57	F	Stop-smoking practitioner	2–5 years
P6 FG	56	F	Stop-smoking-in-pregnancy specialist	>10 years

## Data Availability

The data presented in this study are available on request from the corresponding author. The data are not publicly available as consent was not obtained to hold them in a public repository.

## Supplementary Materials

The following are available online at https://www.mdpi.com/article/10.3390/ijerph18094673/s1, Figure S1: comparison coding tree.
