# Supplementary figures and images for: Agreement and Disparities between Women and Stop-Smoking Specialists about How to Promote Adherence to Nicotine Replacement Therapy in Pregnancy
Source: Int J Environ Res Public Health. 2021 Apr 28;18(9):4673. doi: 10.3390/ijerph18094673 (PMC8125676; doi:10.3390/ijerph18094673)

**SM Figure 1: Coding tree**

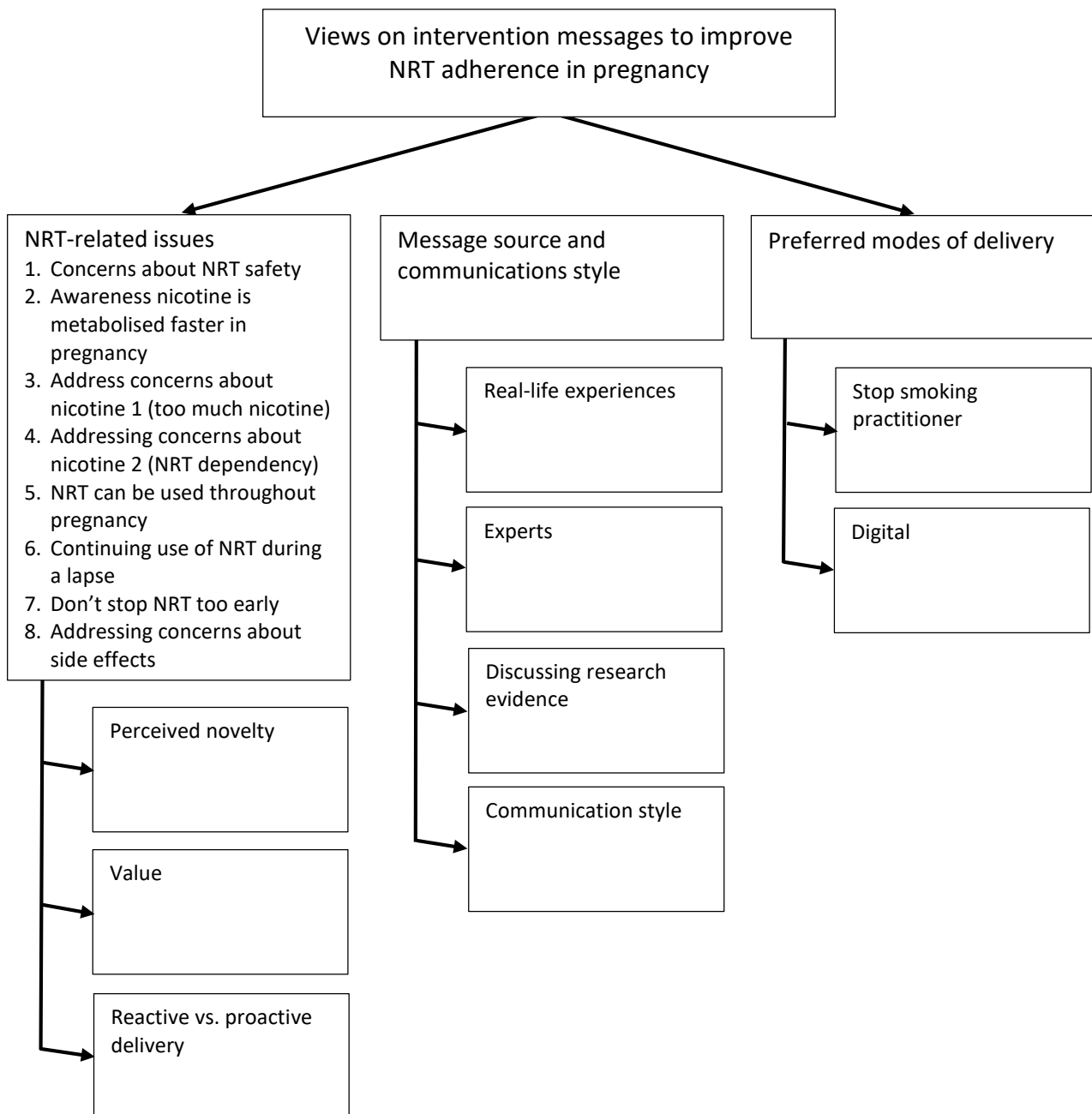

Supplement: Supplementary file 1 [file ijerph-18-04673-s001.zip › ijerph-1176263-supplementary.pdf]
